# Characteristics of Dissolved Organic Matter Impacted by Different Land Use in Haihe River Watershed, China

**DOI:** 10.3390/ijerph20032432

**Published:** 2023-01-30

**Authors:** Zhaochuan Chen, Yanan Wen, Min Xiao, Fujun Yue, Wenxi Zhang

**Affiliations:** 1 Tianjin Key Laboratory of Water Resources and Environment, Tianjin Normal University, Tianjin 300387, China; 2 Institute of Surface-Earth System Science, School of Earth System Science, Tianjin University, Tianjin 300072, China; 3 Haihe Laboratory of Sustainable Chemical Transformations, Tianjin 300192, China

**Keywords:** dissolved organic matter, Haihe River, EEM-PARAFAC analysis model, land use, source distribution, seasonal variations

## Abstract

It is important to explore characteristics of dissolved organic matter (DOM) in the riverine system due to its critical role in the carbon cycle. This study investigated the distribution characteristics and sources of DOM based on excitation emission matrix three-dimensional fluorescence technology and parallel factor (EEM-PARAFAC) analysis at two rivers in northern China strongly impacted by human activities. The results show that the fluorescence intensity of terrestrial humic-like substances increased during summer in Haihe River. The intensity was significantly higher than in spring due to terrestrial detritus from runoff conveyance. The fluorescence intensity of protein-like substances in spring was the highest and decreased in summer. This feature of DOM in the Duliujian River was related to the increase in precipitation and surface runoff in the wet season and the rapid degradation of mixed DOM in the dry season. An analysis of HIX, BIX and FI showed a low degree of DOM humification and more endogenous contributions from microbial and phytoplankton degradation. Seasonal variations of dissolved organic carbon (DOC) and chromophoric DOM (CDOM, a_335,_ thereinto C1) suggest that chromophores, particularly terrestrial substances, regulate the temporal patterns of DOM in the two rivers. Combined with the analysis of the proportion of land use types in riparian buffers, tillage had a great impact on DOM content and hydrophobicity in Haihe River watershed. Domestic wastewater and industrial sewage discharge contribute more DOM to Duliujian River watershed, which was indicated by more abundant protein-like components (212.17 ± 94.63 QSU in Duliujian River;186.59 ± 238.72 QSU in Haihe River). This study highlights that different land use types resulted in distinctive sources and seasonal dynamics of DOM in rivers. Meanwhile, it should be considered that the estimation of carbon cycling should involve monitoring and evaluating anthropogenic inputs into rivers.

## 1. Introduction

Dissolved organic matter (DOM), an indispensable component of organic compounds in aquatic ecosystems, originates from bacteria, phytoplankton and macrophytes, and is produced by photochemical decomposition and microbial processes [1,2]. DOM also plays an important role in the carbon biogeochemical cycle and water quality evolution of aquatic systems, reflecting biogeochemical processes by describing water quality characteristics, nutrients, pollutants and the optical properties of the water environment [3]. In recent years, rapid urban development and high-intensity agricultural planting activities in basins have produced a large number of pollutants discharged into rivers, lakes and other waters, resulting in increasing water pollution and a series of ecological and environmental problems, making the detection and evaluation of surface water environmental quality extremely important.

With the wide application of spectral technology, ultraviolet-visible (UV-Vis) absorption spectroscopy and fluorescence spectroscopy have been used as effective tools for characterizing the spectral properties of DOM [4,5,6]. Chromophoric dissolved organic matter (CDOM) is the largest reservoir of dissolved organic carbon (DOC) in water environments and the main light-absorbing substance in natural DOM reservoirs [7], which can be measured by UV-Vis spectrum [6]. Due to several advantages, CDOM characterization measures such as the three-dimensional fluorescence spectroscopy (3-EEM) technique can be used to rapidly and inexpensively identify fluorescent components and pollution sources, which is particularly suitable for surface water restoration where it is consistently polluted by complex sources and where current water quality parameters are insufficient for effective evaluation [8]. Fluorescence excitation–emission matrix combined with parallel factor analysis (EEM-PARAFAC) has been utilized to determine if the optical indices of DOM can obtain effective information on organic substances and explain organic composition and dynamic factors in different environments [9,10,11]. The chemical structure characteristics of DOM in water mainly include allochthonous inputs from soil and litterfall and autochthonous inputs from algae and microbial activities [12]. Organic pollution makes DOM structure and the transformation process of water more complicated, which affects the surface water environmental quality of the basin. The analysis of spectroscopic techniques has a great influence on the study of DOM in natural ecosystems and is conducive to predicting the behavior changes of environmental pollutants [13].

Land use is one of the important factors that affect the composition of DOM in a watershed, so it is feasible to estimate the distribution characteristics of DOM by land classification [14,15]. In view of the obvious differences in water pollution in different river basins, researchers have studied the characteristics of DOM abundance and fluorescence components in different riparian land use types, and proven that human activities such as urban construction, industrial development and agricultural production have had a significant impact on the total amount and composition of DOM in river basins [16,17,18]. The effects of land use type on DOM in river water are varied [19], but there are few studies on the coupling of DOM seasonal variation and land use/cover change.

Land cover has an important effect on organic matter sources, composition and content in aquatic environments which are involved in water quality change by physical migration and chemical decomposition, as well as biological action [20]. Water quality parameter (EC, SAL, etc.) changes are caused by soil denudation and mineralogical ingredient dissolution in water. Values of pH and DO are used to refer to primary productivity, photosynthesis and degradation, which is an endogenous metabolism of organic matter and generally produces micro-molecules of relatively simple structures [21,22]. Terrestrial components by runoff and seepage usually have characteristics of macro-molecules and complex structures. Therefore, the study of seasonal land use has significant implications for land-use planning and policy recommendations. In this study, the rivers of the Haihe and Duliujian River watershed in Northern China were sampled to analyze the optical properties, sources, and spatiotemporal distribution of DOM in water. The research purposes are as follows: using UV-Vis spectroscopy and EEM-PARAFAC models to track the source and analyze changes of DOM components under different seasonal and hydrological conditions, exploring the spatiotemporal distribution of organic components and the influencing factors; explaining the impacts of land use and human activities on DOM by analyzing land cover types and spectral indices in different seasons, and providing a reference for water environment quality evolution.

## 2. Materials and Methods

### 2.1. Site Description

Haihe River is located in Northern China, and the lower reaches after the confluence of Tianjin is called the Haihe River, forming the Haihe River Basin (112°~120° N, 35°~43° E). The study objects are the trunk streams of Haihe River and Duliujian River in Tianjin. Haihe River flows through Tianjin, from the confluence of Ziya River and North Canal in the west to the tidal gate of Haihe River in the east, with a total length of 76 km. The river network in this section is densely covered, with natural and artificial rivers crisscrossing. Duliujian River belongs to the Haihe River system, which is the largest tributary in the lower reaches of the southern Haihe River system. In the upstream catchment, it is composed of different tributaries (Ziya River, Daqing River and South Canal), and the artificially excavated channel for flood discharge, with a total length of 67 km.

This area belongs to the temperate monsoon climate zone, which is affected by the Mongolia continental air mass and the Pacific Ocean. It is a semi-humid and semi-arid region with an average annual temperature of about 12 °C, an average annual precipitation of 540~700 mm, and a water surface evaporation of 1100 mm. The rivers in the study area are mainly used for fault drainage, flood discharge, urban backup water resources, agricultural irrigation and industrial water [23].

### 2.2. Sample Collection and Water Quality Testing

**Figure 1:**
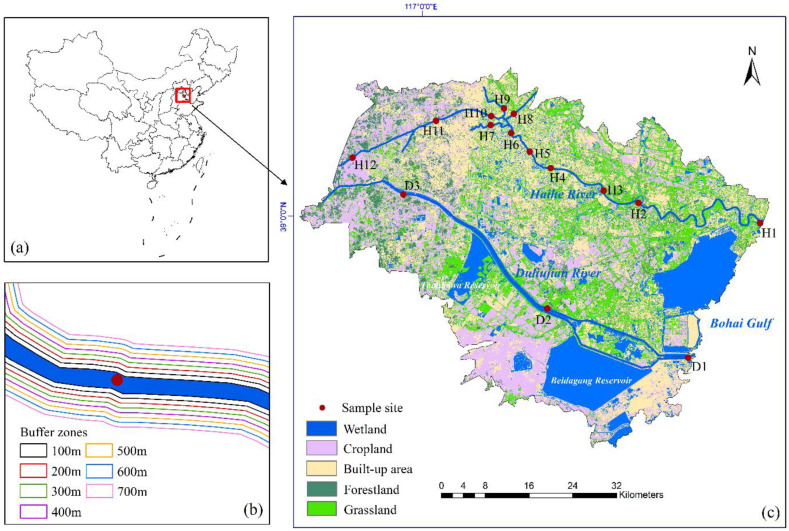
(**a**) The location of the study area in China; (**b**) Sampling sites and land use types of Haihe River and Duliujian River; (**c**) Riparian buffer zones with different widths at sampling points. ‘H’ denotes Haihe River, ‘D’ denotes Duliujian River.

A total of 15 sampling sites (H1–H12, D1–D3) was set up in the Haihe River and Duliujian River, and surface water was collected once a month from March to December in 2021 (spring is from March to May; summer is from June to August; September to November count as autumn; December as winter) (Figure 1).

H1–H6 were distributed in the main stream section of Haihe River, H7, H8 and H9 were located in the main tributaries of the Haihe River in the South Canal, Xinkai River and North Canal, respectively, H10, H11, H12 were located in the tributaries of the Ziya River, and D1–D3 were collected in different sections of Duliujian River. An In-Situ Aqua TROLL 600 multi-parameter water quality monitor was used to measure basic parameters such as water temperature (T), pH, dissolved oxygen (DO) and electrical conductivity (EC) in the field. Samples were collected in acid-washed brown polyethylene bottles after being pre-rinsed with sample water. To avoid pollution, all sample bottles were soaked with 10% HNO_3_ for 24 h in advance and rinsed with distilled water. The water sample was filtered through a 0.45 µm glass fiber membrane (burned at 450 °C for 5 h), then placed in a refrigerator at 4 °C for subsequent analysis.

DOC was determined by high temperature catalytic oxidation method (Aurora 1030 W TOC). Milli-Q water (18.2 MΩ∙cm) was used as a blank, and potassium hydrogen phthalate solution was used as a standard reagent. The relative standard deviation (RSD) of repeated measures was less than 5% [24].

### 2.3. UV-Vis Absorption Spectra and Fluorescence Spectroscopy Analysis

The chilled water samples were warmed to room temperature (20 °C) overnight prior to spectral analysis. The UV-Vis absorption spectrum was measured between wavelengths of 200 nm and 700 nm at intervals of 1 nm using a T9cs spectrophotometer (Persee, China) in a 1-cm cuvette. Absorbance at each wavelength (λ) was baseline-corrected by subtracting the absorbance of Milli-Q water (18.2 MΩ∙cm). The absorbance A(λ) of the measured sample was calculated into the uncorrected CDOM absorption coefficient a(λ′) by Equation (1). Then the baseline drift caused by scattering was corrected according to Equation (2), and the corrected absorption coefficient a(λ) was obtained by the absorption coefficient at 700 nm a(700′).

(1) $$a\lambda^{\prime }=2.303\;\times \;A\left(\lambda \right)/\gamma$$

(2) $$\;a\lambda =a\lambda^{\prime }-\frac{a700^{\prime }\times \lambda }{700}$$

where γ is the path length of the cuvette in meter; A(λ) is the absorbance at wavelength λ, a(λ′) is the uncorrected CDOM absorption coefficient at wavelength λ, and a(λ) is the corrected CDOM absorption coefficient at wavelength λ.

The specific ultraviolet absorption coefficients usually include a_355_, a_260_, SUVA_254_, E_2_/E_3_(a_250_/a_365_) and S_R_, etc.; a_355_ indicates the content of CDOM [25], and a_260_ refers to the content of hydrophobic CDOM containing aromatic C moiety [26]. SUVA_254_ represents the absorbance at 254 nm per unit organic C and has a positive correlation with aromaticity, which is calculated by Equation (3) [27]. The ratio of a_250_/a_365_ implies the relative proportion of fulvic acid and humic acid in DOM and the abundance of humic substances [28]. The spectral slope (S_R_) of the absorption curve is the slope ratio between 275–295 nm and 350–400 nm [25].

(3) $${\mathrm{SUVA}}_{254}{=a}_{254}/\mathrm{DOC}$$

The fluorescence spectroscopy was measured using an F-7000 fluorescence spectrophotometer. The sample was poured into a 1 cm quartz cuvette, and the scanning range was set at excitation wavelength (Ex) 220~450 nm, emission wavelength (Em) 280~550 nm. Scanning speed was 1200 nm·min^−1^, fluorescence readings were collected at intervals of 5 nm excitation wavelength and 1 nm emission wavelength. Milli-Q water was used as a blank, and the fluorescence data of the blank were subtracted from the sample to eliminate the internal filtration effect. The fluorescence intensity was calibrated to fluorescence normalization with quinine sulfate standard solution (1 μg·L^−1^ = 1 QSU (quinine sulfate unit)) at the wavelength pair of Ex/Em = 350/450 nm [5]. PARAFAC modeling is a three-dimensional multivariate statistical analysis, which is widely used in EEM spectra. By decomposing the fluorescence matrix, individual fluorescence components are separated and quantified according to the fluorescence intensity. We used N-Way Toolbox version 3.1 in MATLAB to execute PARAFAC analysis [29].

Parameters such as humification index (HIX), biological index (BIX) and fluorescence index (FI) can be used to identify the source of DOM and the characteristics of humic substances. The calculation of HIX is based on the ratio of the integral value of the fluorescence intensity at the emission wavelength of 435~480 nm to that at the emission wavelength of 300~345 nm at the excitation wavelength of 255 nm. When HIX > 6, DOM has strong terrestrial characteristics; when HIX < 4, the source of DOM is mainly autochthonous [30]. BIX is the ratio of fluorescence intensity at 380 nm to 430 nm under the excitation wavelength of 310 nm. When BIX > 1, DOM is dominated by biological source; for BIX < 0.7, a terrestrial source is dominant [31]. FI is calculated as the ratio of fluorescence intensity at 450 nm and 500 nm emission wavelengths at 370 nm excitation wavelength to distinguish autochthonous and terrestrial sources of DOM. When FI is closer to 1.4, DOM is mainly of terrestrial source; when FI approaches 1.9, DOM is mainly of autochthonous source [32]. Higher BIX and FI are usually related to higher levels of DOM microbial, planktonic and algal sources.

### 2.4. Land Use Classification and Statistics

The watershed map of the study area was made by watershed extraction on the basis of a digital elevation model (DEM, 30 m) using the analysis tool in ArcGIS 10.2. Then, according to remote sensing images (Landsat 8, Geospatial Data Cloud), ENVI 5.1 was used to cut the scope of the study area, and the areas of interest were selected and underwent supervised classification to make a land-use type map. According to satellite images, the study area was divided into five land types: forestland, cropland, grassland, built-up area and wetland. The riparian buffer zones of sampling sites (with widths of 100 m, 200 m, 300 m, 400 m, 500 m, 600 m and 700 m, respectively and lengths of 1000 m) were established by building the buffer zone in ArcGIS. As the buffer zones were larger, the adjacent buffer zones began to overlap, so the maximum width of 700 m was set, the effective buffer zone range was determined, and the area proportions of different land types in each buffer zone where sampling sites were located was calculated.

The water quality parameters, spectral indexes and fluorescence intensity of DOM components of rivers were analyzed by single factor analysis of variance (ANOVA test) with IBM SPSS 25. Canoco 5 was used to perform redundancy analysis (RDA) between DOM indexes and land-use proportions; their relationships in different seasons were analyzed and expressed with Pearson correlation coefficients.

## 3. Results

### 3.1. Characteristics of Water Quality Parameters

The water quality parameters of Haihe River and Duliujian River in different seasons are shown in Table 1. The pH values of both rivers, depending on hydrogen concentration, ranged from 5.81 to 10.82 and 7.97 to 9.53, respectively, with average values of 8.42 and 8.58, indicating that the river water was weakly alkaline during the study period, and there was no significant seasonal change. Rivers are the carriers of pollution, discharge of sewage, dirt-washing and dipping industrial materials, and these are the sources of alkalinity. Soil leaching, the level of dissolved inorganic carbon and biomass directly affect the pH of river water. Spatially, the pH of the lower reaches of Haihe River was higher than that of the middle and upper reaches, while the pH showed little change across Duliujian River. The pH result is higher than that (6.96 ± 0.46) in Juru River, Malaysia, where it was influenced by habitat degradation and pollution [33]. For DO, the seasonal variation trend of Haihe River and Duliujian River was the same, with the highest in winter (mean: 14.17 ± 2.44 mg·L^−1^ and 14.01 ± 0.05 mg·L^−1^, respectively) and the lowest in summer (mean: 6.76 ± 3.08 mg·L^−1^ and 7.78 ± 1.88 mg·L^−1^, respectively).

There were significant differences in DO between winter and summer (*p* < 0.05). The electrical conductivity (EC) of the two rivers was quite different, and the EC fluctuation of Haihe River was small, with an average value of 1.7 ± 2.68 ms·cm^−1^, which was lower than that of Duliujian River (7.88 ± 15.22 ms·cm^−1^). For Duliujian River, the average value of EC reached 22.54 ± 22.4 ms·cm^−1^ in spring, which was significantly higher than for other seasons. The average concentration of chlorophyll-a (Chl-a) in Haihe River was the highest in autumn (53.91 ± 47.99 μg·L^−1^) and the lowest in spring (20.76 ± 23.58 μg·L^−1^), and there was a significant difference between the two seasons (*p* < 0.05). However, the average value of Chl-a in Duliujian River was the highest in summer and the lowest in winter, with average values of 34.98 ± 22.17 μg·L^−1^ and 18.59 ± 3.5 μg·L^−1^, respectively. The annual average values of H1 and H12 were significantly higher than at other sampling sites, and H12 was the highest with an average value of 100.55 ± 82.39 μg·L^−1^, indicating that the Chl-a of different reaches of Haihe River varied greatly.

Cl^−^ and SO_4_^2−^ accounted for a relatively high proportion of anions in the two rivers, with an average annual proportion of more than 80%, and Na^+^, Mg^2+^ and Ca^2+^ contributed highly to cations, while the concentration of Ca^2+^ in Duliujian River was lower than that in Haihe River. The HCO_3_^−^ concentration ranged from 1.42 to 8.07 and 2.71 to 1.80 mmol/L in Haihe and Duliujian River, respectively.

### 3.2. Seasonal Variation of DOC and CDOM Absorption Coefficients

**Figure 2:**
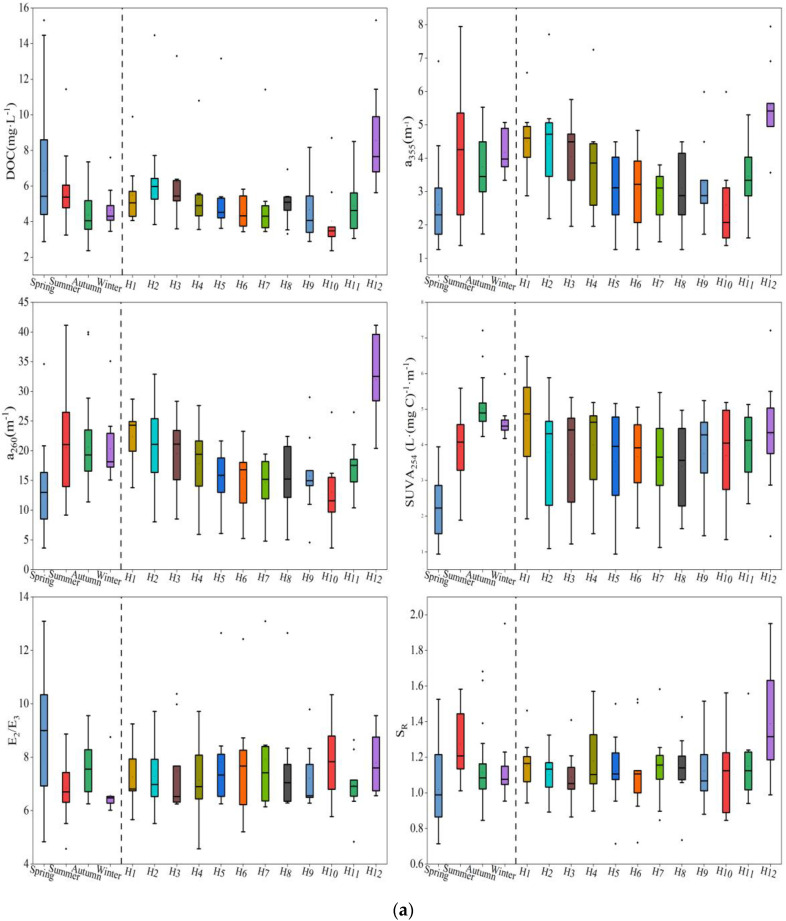

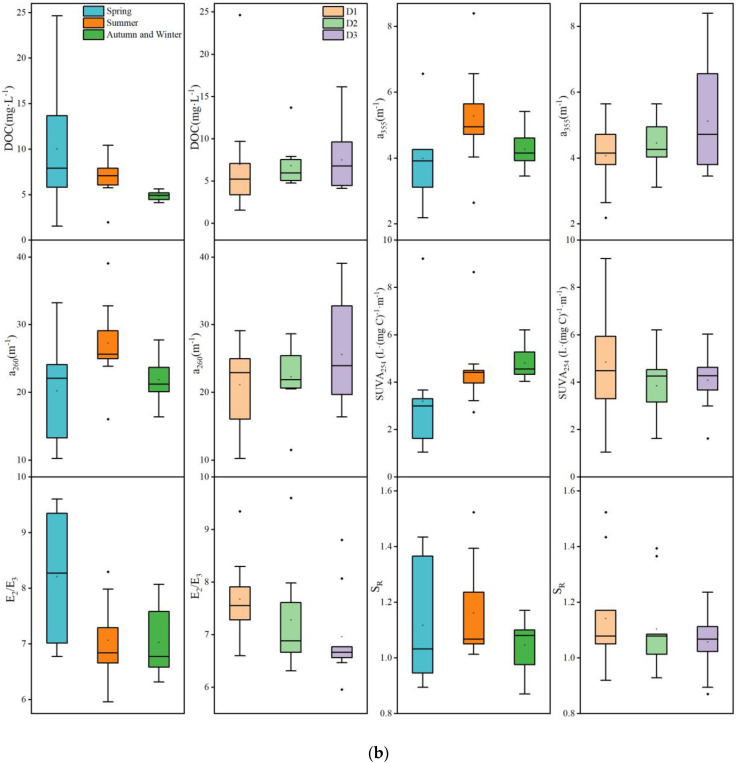
Temporal and spatial variation of DOM parameters (DOC, a_355_, a_260_, SUVA_254_, E_2_/E_3_ and S_R_) in Haihe and Duliujian River: (**a**) in Haihe River; (**b**) in Duliujian River. (The black line and hollow square in boxes, lower and upper edges, bars and dots outside the boxes represent median and mean values, 25th and 75th, 5th and 95th, and <5th and >95th percentiles of all data, respectively).

The seasonal variations of dissolved organic carbon (DOC) in the two rivers were significant (Figure 2).

The DOC concentration ranges of Haihe River and Duliujian River were 2.37~15.31 mg·L^−1^ and 1.54~24.64 mg·L^−1^, respectively. The average values of DOC in Haihe River and Duliujian River were 5.46 ± 2.35 and 7.08 ± 4.53 mg·L^−1^, respectively, lower than those in other major rivers and lakes in northern China, e.g., 138.6 mg·L^−1^ in Harbin and 10.71 mgL^−1^ in Beijing [34]. DOC in spring was obviously higher than in other seasons (*p* < 0.01) for both rivers, with average values of 6.84 ± 3.42 mg·L^−1^ in Haihe River and 10.04 ± 7.15 mg·L^−1^ in Duliujian River. The values for Duliujian River were higher than those for Haihe River, and the trend was the same in other seasons. In addition, comparing with other sampling sites in Haihe River, DOC in H12 was the highest in every month, with an annual average of 8.55 ± 2.94 mg·L^−1^; the maximum value appeared in March (15.31 mg·L^−1^). Except for H12, the concentration of DOC in the lower reaches of the Haihe River was generally higher than that in the upper reaches.

The absorption coefficient a_355_ of CDOM in Haihe River was higher in summer and winter, with average values of 4.09 ± 1.82 m^−1^ and 4.17 ± 0.64 m^−1^, respectively, and the lowest value of 2.6 ± 1.27 m^−1^ was measured in spring, which is much lower than in the other three seasons (*p* < 0.05). However, the average value of a_355_ in Duliujian River was 4.55 ± 1.28 m^−1^, which was superior than that for Haihe River (3.66 ± 1.45 m^−1^), and the highest value was measured in summer (5.28 ± 1.61 m^−1^). The average value of a_260_ in Haihe River was the lowest in spring (12.93 ± 6.07 m^−1^), while it differed little between the other three seasons. Duliujian River had the highest a_260_ in summer (27.28 ± 6.36 m^−1^) and the lowest value in winter (19.11 ± 2.39 m^−1^). These variations of a_355_ and a_260_ showed similar seasonal distribution patterns from upstream to downstream in the two rivers.

SUVA_254_ is used to characterize the aromaticity of DOM. The aromaticity changes of Haihe and Duliujian River were very similar. Both had the lowest values in spring, with averages of 2.22 ± 0.85 and 3.2 ± 2.43 L·(mg C)^−1^·m^−1^, respectively, and rapidly escalated to 3.95 ± 0.84 and 4.57 ± 1.67 L·(mg C)^−1^·m^−1^ in summer. SUVA_254_ reached a maximum in autumn in each river (5.02 ± 0.58 and 5.06 ± 0.75 L·(mg C)^−1^·m^−1^) and decreased in winter. The average values of SUVA_254_ at the sampling sites H1 and D1 were the highest (4.68 ± 1.38 and 4.85 ± 2.52 L·(mg C)^−1^·m^−1^, respectively).

The average values of E_2_/E_3_ (a_250_/a_365_) for the two rivers were highest in spring, with 8.96 ± 2.34 and 8.22 ± 1.21, respectively, and lowest in winter, with 6.58 ± 0.7 and 6.64 ± 0.04. The seasonal variation of E_2_/E_3_ in Duliujian River was not obvious, and the average value for Haihe River in spring was significantly different from those in summer and winter (*p* < 0.05). Overall, the E_2_/E_3_ of the upper reaches of Haihe River was higher than that of the lower reaches, but the situation of Duliujian River was the opposite. S_R_ for Haihe River ranged from 0.71 to 1.95, and the values for Duliujian River ranged from 0.87 to 1.52. The average values of S_R_ in summer were the highest in both rivers, 1.27 ± 0.18 and 1.16 ± 0.18, respectively, and the value in Haihe River in summer was significantly different from those in spring and autumn (*p* < 0.01).

### 3.3. Fluorescence Spectroscopy Analysis by PARAFAC Modelling

**Figure 3:**
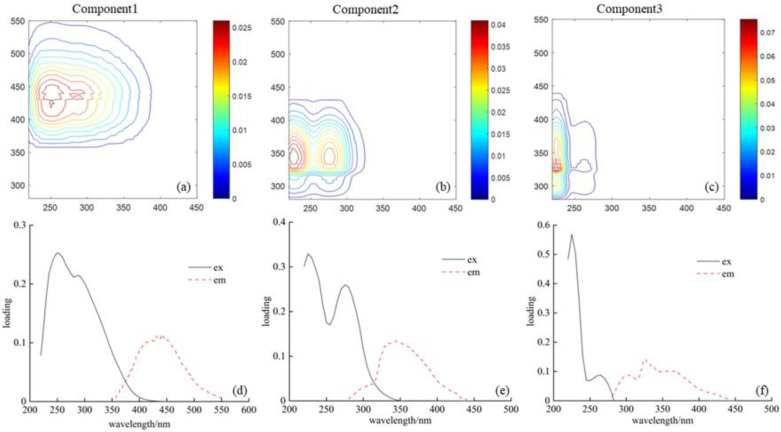
The PARAFAC model output showing fluorescence signatures of the three fluorescent components. (**a**–**c**) The contour plots for C1, C2 and C3, respectively; (**d**–**f**) The excitation and emission loadings for C1, C2 and C3, respectively.

The three fluorescent components are depicted in Figure 3.

Component type and peak position were compared with previous data in various environments, as shown in Table 2. Component 1 (C1) showed two fluorescence peaks (peak A: Ex/Em = 230~260/400~449 nm, and peak C: Ex/Em = 285~310/400~449 nm), which were similar to the terrestrial humic-like substances defined in some surface water studies [4,35,36] (Figure 3a).

The humic-like component appeared in four seasons for Haihe River; the intensity of peak A was higher than that of peak C, and peak C only appeared from March to June. For Duliujian River, the C1 component disappeared in May and December. Component 2 (C2) showed two fluorescence peaks (peak Tuv: Ex/Em = 225~235/340~350 nm, peak T: Ex/Em = 275~290/337~350 nm), usually classified as tryptophan-like substances in protein, and peak T was weaker than peak Tuv (Figure 3b). Tryptophan-like fluorophore is structurally unstable, which is related to the aromatic protein-like structures produced by microbial degradation, and have been reported in other studies of surface water [16,34]. This fluorescent substance was not detected in the Duliujian samples in September, while it was present in water samples of Haihe River perennially. Component 3 (C3) was a tyrosine-like substance with two fluorescence peaks (peak Tuv: 225/300~327 nm, peak T: 260~265/300~327 nm), which was determined to be autochthonous tyrosine-like in protein substances [35] (Figure 3c). Such substances only appeared in March and April in Haihe River and was associated with the degradation of phytoplankton.

The average fluorescence intensities of all samples in Haihe River and Duliujian River were 120.35 ± 137.7 and 178.15 ± 91.76 QSU, respectively. The fluorescence intensity of each component in Duliujian River (C1: 139.87 ± 72.76 QSU, C2: 212.17 ± 94.63 QSU) was generally higher than in Haihe River (C1: 75.81 ± 31.45 QSU, C2: 168.04 ± 180.08 QSU, C3: 104.07 ± 146.79 QSU). The fluorescence intensity of C1 in Haihe River was the highest in summer (mean: 85.51 ± 34.68 QSU) and the lowest in spring (mean: 66.23 ± 36.38 QSU), while the fluorescence intensity of C2 was the highest in spring (mean: 197.64 ± 291.99 QSU), 3 times above humic moiety, accounted for by 75% fluorophores and 44.09 L QSU mg^−1^ in DOC. C2 was lower in autumn and winter (mean: 133.66 ± 64.71 and 137.4 ± 53.91 QSU, respectively).

**Figure 4:**
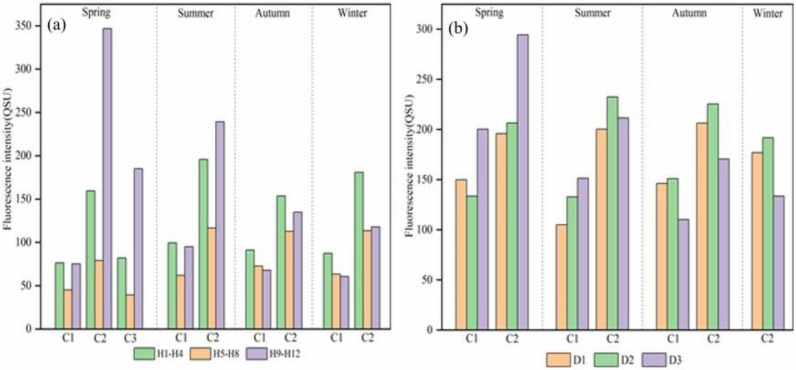
Seasonal and spatial distribution of fluorescence intensity of DOM components (C1, C2 and C3) in two rivers: (**a**) in Haihe River; (**b**) in Duliujian River.

The highest fluorescence intensity of C1 and C2 in Duliujian River appeared in spring. The average values were 161.25 ± 95 and 232.18 ± 108.72 QSU, respectively, and the lowest values appeared in summer (129.67 ± 56.73 QSU) and winter (167.33 ± 30.29 QSU). In addition, the protein-like substances in Haihe River in spring were obviously different from those in the other three seasons. Tryptophan-like substances (C2) did not change during the whole year, and C2 was highest in spring (2341.12 QSU). C3 was only detected in the spring samples, which was identified as a native tyrosine-like substance. The total fluorescence intensity of protein-like substances in Haihe River in spring was the highest (3567.17 QSU), with a fluorescence intensity that was much higher than in other seasons (Figure 4).

**Figure 5:**
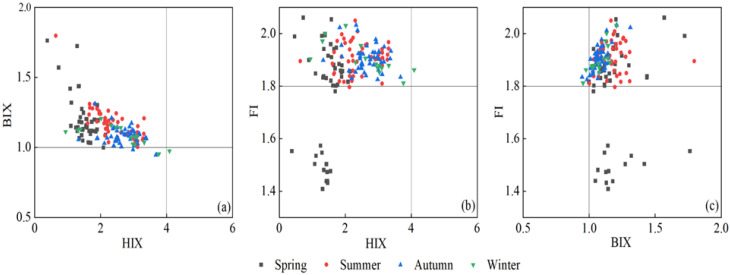
Seasonal variation of the humification index (HIX), biological index (BIX) and fluorescence index (FI) in Haihe River and Duliujian River. (**a**–**c**) are the correlation figures between HIX and BIX, HIX and FI, BIX and FI.

The humification index (HIX) of Haihe River ranged from 0.38 to 4.09, and that of Duliujian River ranged from 0.89 to 3.31, their annual average values were lower than 5 (Figure 5a,b), indicating that the source of DOM in both rivers was mainly autochthonous.

The quarterly average of HIX in Haihe River increased successively from spring to winter, while the value in Duliujian River was much higher in summer and autumn than in winter and spring (*p* < 0.01). In general, HIX in the upstream region of Haihe River was lower than that in the middle and lower reaches, while HIX in the middle reaches of Duliujian River was the highest. The biological index (BIX) values of Haihe River and Duliujian River ranged from 0.95 to 2.24 and 1 to 2.27, respectively, with an average of 1.15 ± 0.16 and 1.19 ± 0.24 (BIX > 1), showing strong signs of biogenesis. HIX and BIX were 0.3 and 2.4 times those reported in [38]. The maximum of Haihe River was at H12 in spring, while BIX at H9 and H10 in winter were less than 1 (0.95 and 0.98), which reflects the positive influence of low temperature on inert microbial activities. The average FI of both rivers was mostly close to 1.9 (Figure 5b,c).

### 3.4. RDA Models: Environmental Predictors versus the Quantity and Quality of DOM

Of the sampling sites in the study area (Figure 1), H1, H2, H3 and H4 had a prominent area proportion of grassland and built-up land, and the proportion of built-up area gradually increased from downstream to midstream. H5, H6, H7, H8, H9 and H10 were located in urban areas, where man-made construction was pervasive. Upstream, the proportion of cropland and forestland increased at H11 and H12. D1 had the highest proportion of built-up area, D2 was dominated by grassland and built-up area, and cropland accounted for the highest proportion at upstream D3. The proportion of land-use types around each sampling site varied greatly from the upstream to the downstream areas, and substance circulation will take place with climatic change and anthropic intervention.

Significant spatial variations in DOM quantity and quality were observed in Haihe and Duliujian River, and some variations can be directly linked to forestland and cropland use. In Haihe River, for the DOM quantity and quality model with dependent variables as DOC, and fluorescence intensity of three fluorescence components (C1, C2 and C3), S_R_, a_260_, two axes (RDA1 and RDA2) of the redundancy analysis together explained 69.91%, 61.16%, 59.44% and 68.48% of the total variance in spring, summer, autumn and winter, respectively. RDA1 accounted for the majority (63.39%, 48.21%, 52.22%, 55.49%) of the variance, and RDA2 explained only 6.52%, 12.95%, 7.22% and 12.99%.

The monitoring data for Duliujian River watershed show that agricultural land use amplified the amount of DOM in rivers, as evidenced by overall concentrations of DOC in spring, summer and winter. For the redundancy analysis, two axes (RDA1 and RDA2) together explained 100% of the total variance in the four seasons. RDA1 accounted for the majority (81.06%, 67.94%, 77.99%, 80.14%) of the variance, and RDA2 explained only 18.94%, 32.06%, 22.01% and 19.86%.

## 4. Discussion

### 4.1. Source Analysis of DOM

There are generally two sources of DOM in water, namely terrestrial and autochthonous sources. Terrestrial source mainly consist of humus formed by the degradation of terrestrial plants or soil organic matter, which is washed into water by runoff or rainfall. Autochthonous sources are mainly generated by the degradation of phytoplankton by bacteria and light radiation [12]. In Haihe River and Duliujian River, a_355_, representing CDOM content, had a significant linear fit with DOC concentration in summer, autumn and winter (*p* < 0.01) (Figure 4), while the regression was poor in spring, which reflects a discrepancy between the sources and sinks of DOM. CDOM could be used to represent DOM to exploit the variations. Therefore, in summer, autumn and winter, the DOM and CDOM in Haihe River and Duliujian River had the highest concentrations in summer, due to more precipitation, strong light, rapid degradation of phytoplankton and intense microbial activities, which increased DOM mainly from endogenous production and allochthonous input. However, the a_355_ of Haihe River was also high in winter, which was due to the lower temperature which inhibited the microbial usage of protein-like substances; this result is consistent with [8]. Low temperatures and short light duration slowed down the photodegradation rate of DOM, which favored the preservation of humic-like substances.

In Haihe River, there was a significant linear correlation between CDOM (a_355_) and the fluorescence intensity of C1(R^2^ = 0.624, *p* < 0.01), a_355_ and C2 (R^2^ = 0.345, *p* < 0.01), as well as the correlation between a_355_ and C1 in Duliujian River in summer and autumn (R^2^ = 0.269, *p* < 0.05). This suggest an important role of humic-like/tryptophan-like moieties in deciding the temporal variation of DOM in both rivers, attributed to natural and anthropic sources, which is consistent with previous studies [13,14]. C1 and C2 in individual river water samples exhibited a similar spatiotemporal pattern (R^2^ = 0.57, *p* < 0.01, in Haihe River; R^2^ = 0.768, *p* < 0.01, in Duliujian River) but with fewer seasonal differences in their amount and proportion of total fluorescent intensity, indicating a mixed nature of terrestrial-like and protein-like substances and homology of the two components. The concentration of Chl-a was used to indicate the content of pigment and phytoplankton, with a strong positive correlation between DOC and chlorophyll-a in autumn and winter (R^2^ = 0.542 and R^2^ = 0.724, *p* < 0.01), indicating that the organic matter synthesis and degradation process in Haihe River showed large seasonal differences. The product of photosynthesis of aquatic plants partially constituted the autogenic source of DOM (chlorophyll-a *vs* C1/C2, R^2^ = 0.383, 0.364, *p* < 0.05). Microbial humic-like DOM in Haihe River was sourced primarily from soils because its relative abundance was positively correlated with cation concentrations and conductivity (R^2^ = 0.446 for Ca^2+^; R^2^ = 0.492 for Na^+^; R^2^ = 0.495 for EC; *p* < 0.01), which are two indicators of soil inputs to streams [15]. Land covers differ in different seasons made human operation changed, and afflux of organic components affected water quality in the watershed [15,39]. Therefore, the protein-like substances in Haihe River may be produced by native phytoplankton sources and metabolic activities of macrophytes, and some of them may be decomposed by microorganisms in addition to allochthonous microbial loading with land cover change [5,39,40].

### 4.2. Spatial and Temporal Factors Influencing DOC Concentrations

The variation in DOC concentration may be related to the water quality of the river system, especially in Haihe River. In spring and summer, there was a negative correlation between the DOC level of Haihe River and water temperature (R^2^ = 0.416, *p* < 0.05; R^2^ = 0.144, *p* < 0.01), and there was a significant positive correlation between DO (or pH) and DOC concentration in Haihe River in autumn and winter (R^2^ = 0.243 and R^2^ = 0.635, *p* < 0.01). Hypoxia is very common in summer due to high consumption of dissolved oxygen by microbes and high temperature driving to spill out of water. Therefore, primary production has become a key trigger for nutrient and oxygen dynamics in surface water.

The concentrations of DIC were highest in spring, ranging from 1.45 to 8.43 mmol/L^−1^ (from site H9 and H12) in upper reaches, which is 2.2 times the value at H1. DIC predominantly originated from carbonate weathering but not organic matter mineralization. A high concentration of DIC promoted photosynthesis of aquatic plants and significantly increased the concentrations of Chl a (R^2^ = 0.157, *p* < 0.01). The concentrations of Chl a were shown to have a similar increasing pattern, insofar as the increase between sites H9 and H12 ranged from 0.165 to 288.5 μg∙L^−1^. Carbon dioxide from lithological weathering contributed considerably to algae photosynthesis. The plant-limited nutrient is C, apart from N and *p*, and DIC fertilization is the main reason for DOC formation. DOC, the product of aquatic plant photosynthesis, exhibited a significant linear correlation with DIC in autumn and winter, which indicates that a DIC fertilization effect was important in promoting in increase in autochthonous organic matter [41].

It has been reported that tilling activities can mobilize shallow soil DOC to adjacent streams [42]. H12 was located in a large area of cropland, where frequent farming practices and hydrological modifications resulted in high concentration of DOC. Intensive drainage systems widely used have been reported to increase the riverine DOC flux, but this is the short-term effect. Long-term export is supposed to decrease as a result of discharge reduction due to stronger evapotranspiration and reduced infiltration, and subsurface water extraction for irrigation, which were responsible for relatively low concentrations of DOM in autumn and winter in both rivers [43].

**Figure 6:**
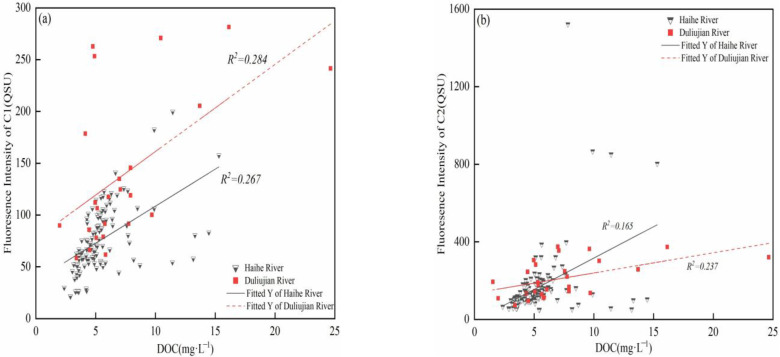
Correlations between dissolved organic carbon (DOC) concentration and the fluorescence intensity of components in Haihe River and Duliujian River: (**a**) DOC and C1; (**b**) DOC and C2.

The relationship between DOC concentration and fluorescence signals is complex, and DOC is affected by precipitation, soil type, sewage discharge, algae and other factors [37]. The identified PARAFAC components are good indicators for tracing pollutant sources in freshwater. To further explore the influence of land-use changes and seasons on the DOM characteristics in the watershed, the correlation between PARAFAC components and DOC is analyzed in Figure 6.

Linear regression was conducted separately according to land use (i.e., agricultural in the upstream sites, urban in the downstream sites) and season. For terrestrial component C1, a strong correlation (R^2^ = 0.267, *p* < 0.01; R^2^ = 0.284, *p* < 0.05) was observed in both rivers throughout the year. This indicates that the accumulative terrestrial inputs such as non-point sources (NPS) constitute a major part of the pollution loading of Duliujian and Haihe River in the agricultural region [44]. Therefore, the water restoration strategy should focus on NPS control. The significant accumulation of humic-like and protein-like (R^2^ = 0.534, *p* ≤ 0.01) substances in the downstream area was a distinguishing characteristic between agricultural and urban regions; therefore, terrestrial substances explained the variation of DOC along the DOC in the downstream region of the river to a lesser extent (Figure 4). An exception is that C2 at H12 in a cropland use region was almost 8.4 times higher than downstream in spring.

The correlation between DOC and C2 in the two rivers (R^2^ = 0.165, *p* < 0.01; R^2^ = 0.237, *p* < 0.01) confirms the influence of intensive human activity in the watershed, with compound fertilizer and pasture manure application as well domestic sewage discharge in ploughing areas and human habitats. C1 and C2 exhibited correlations with DOC in each season in Haihe River, but the correlations become stronger upstream, suggesting that both components contribute a larger proportion of DOM. The correlation analysis demonstrates that Haihe and Duliujian River experience different pollution pressures in the upstream and downstream areas due to the mixed land-use pattern. The correlation between protein-like components and DOC could be an effective tool for evaluating sewage removal performance. If the upstream region in Haihe River showed a similar weak correlation as in the downstream region, the pressure from sewage discharge could be relieved.

The results of FDOM components showed that the highest FDOM was 4354.07 QSU in spring, whereas C1 (terrestrial humic-like) was the lowest in spring (786.89 QSU), accounting for 18.1% of the total fluorescence intensity, the highest in summer (1026.12 QSU), and decreased in autumn and winter. Due to more precipitation in summer, the erosion of precipitation may increase terrestrial humic-like substances in the basin, and C1 content decreased in winter, which may be due to reduced runoff input and its partial degradation [45]. In contrast, Duliujian River lacked C1 in winter, but it was most abundant in spring (483.74 QSU), which may be due to reduced runoff load in winter, inducing almost no terrestrial input and an inability to self-synthesize with microbial assimilation and dissimilation under current hydrological conditions [41]. The non-point source release of pollution in winter could be another reason for the lack of C1 in the Duliujian River watershed [8]. C1 is also the product of the photodegradation of terrestrial macromolecular substances, and showed significant seasonal differences. Therefore, FDOM components in different seasons further highlighted the source of DOM and its biogeochemical changes.

Protein-like substances originate from sewage discharge and microbial substances, and are an important indicator of human activity. Under favorable conditions, substances with low molecular weight can be rapidly utilized by bacteria [46]. The content of C2 decreased in summer and autumn in Haihe River, but its proportion in FDOM or DOC was always higher than C1, which indicates that protein-like substances in water were largely produced through effluent and domestic wastewater discharge and supplied nutrients for microbial activity. Heavy precipitation and strong surface runoff in the wet season diluted substances and reduced the absolute C2 content. The fluorescence intensity of tryptophan-like substances in the upstream H12 sampling site was 1063.84 QSU in spring and 523.32 QSU in summer, accounting for 45.4% and 23.7% of the total fluorescence intensity, respectively. In the midstream region, the content and proportion of C2 decreased significantly, and gradually increased near the sampling sites of H2 and H3 in the downstream region due to aquaculture development.

In autumn and winter, the ratio of C2 in each sampling site was sustained at a stable, low-level state, which resulted from the mixing of water in the downstream and upstream areas of the river system leading to dilution and less autochthonous output. Continually decreasing temperature and water flow, and the high uptake and utilization of micromolecules all sustained the low level of protein-like substances [47]. In Duliujian River, the proportion in FDOM of C1 is higher than that of Haihe River. However, C1 was not detected in winter, when the fluorescence intensity of C2 decreased to the lowest for the whole year (501.99 QSU), with poor transference of soil organic matter by runoff and inert microbial activities.

**Figure 7:**
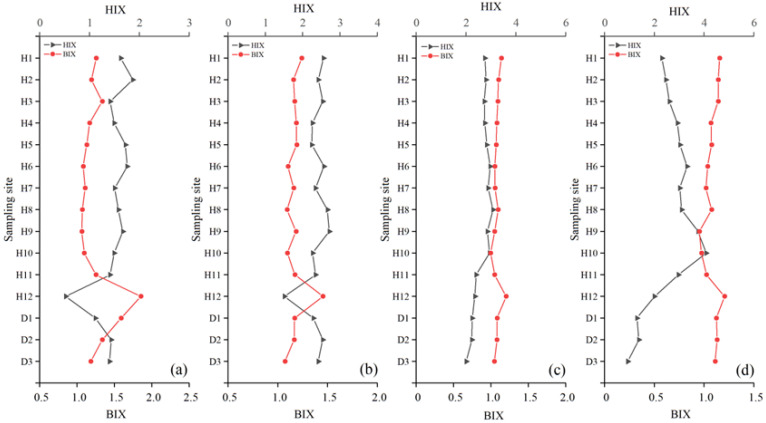
Seasonal variation of the humification index (HIX) and biological index (BIX) on line charts of sampling sites in Haihe River and Duliujian River: (**a**) in spring; (**b**) in summer; (**c**) in autumn; (**d**) in winter. ‘H’ denotes Haihe River and ‘D’ denotes Duliujian River.

By comparing the changes of fluorescence characteristic parameters of all water samples in the four seasons (Figure 7) in Haihe River, it was found that the negative correlation between HIX and BIX was strong in spring and winter (R^2^ = 0.794 and R^2^ = 0.552, *p* < 0.01), mainly due to the preservation of humic substances in rivers under low temperature and poor planktonic/microbial metabolism.

On the other hand, photoradiation on recalcitrant macromolecules was beneficial for breaking the firm structure of macromolecules to form micromolecules and facilitate uptake by microorganisms, further promoted the metabolic activities. There was a strong positive correlation of a_355_ with BIX (*p* < 0.01) in Haihe River, revealing that DOM had a low degree of humification as well as significant autochthonous and bioavailable characteristics. The bioavailability of DOM in spring was the highest, which was the result of manipulation of the agricultural land soil of the radiation zone, with H12 as the core. Likewise, both indexes changed inversely in Duliujian River in spring, but showed a similar spatial trend in autumn and winter. This is due to the fact that the watershed does not potentially have large area of a single land use type and a huge tributary network, and a significant influence of a single source [18]. The variations of individual fluorescent components further elucidate the effects of climatic and hydrological conditions with season on the water quality of Haihe and Duliujian River.

### 4.3. The Impact of Land-Use Types on DOM in Watershed

**Figure 8:**
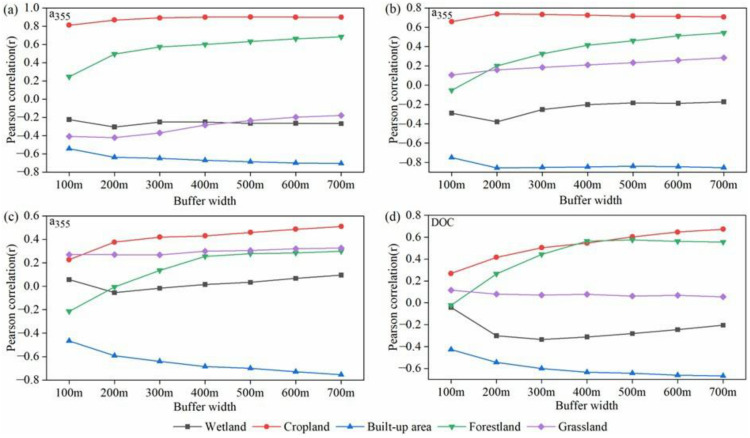
Pearson correlations between chromophoric dissolved organic matter (CDOM) content (a_355_), dissolved organic carbon (DOC) and the various land use coverage in riparian buffer zones with different widths. (**a**) a_355_ in spring; (**b**) a_355_ in summer; (**c**) a_355_ in autumn; (**d**) DOC in winter.

Land use data have shown that cropland, built-up areas and forestland were the main contributors to changes in DOM properties. In spring and summer, cropland and built-up areas were significantly correlated with CDOM. As the buffer width increased from 100 m to 500 m, the correlation between CDOM (a_355_) and cropland increased in spring, and decreased from 500 m to 700 m (Figure 8a). With the increase in buffer width, the significant correlation between built-up area and a_355_ tended to be stable (Figure 8). In winter, the correlation between forestland and DOC reached the highest in the 500 m buffer zone, and then decreased as the width increased (Figure 8d).

Considering the correlation between the changes of DOM indices and the land use types, mainly cropland, built-up areas and forestland, a riparian buffer zone with a length of 1000 m and a width of 500 m was determined as an effective research area.

**Figure 9:**
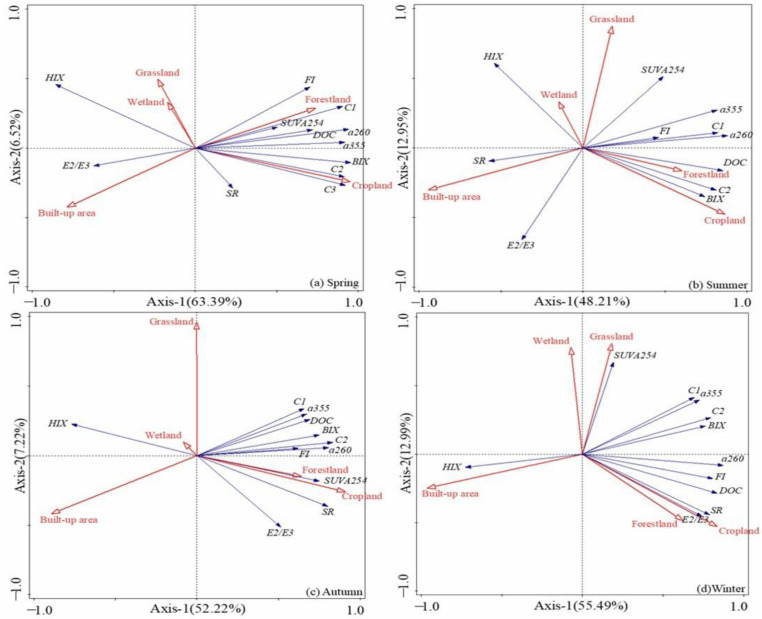
Redundancy analysis (RDA) results of the correlation between land-use types and UV absorption coefficients, fluorescence intensity and fluorescence characteristic parameters (HIX, BIX and FI) in Haihe River.

For Haihe River, DOC, C1, C2 and C3 were aligned better with RDA1 in spring (Figure 9), in which DOC and C1 were positively predicted by the proportion of forestland use, and had strong aromaticity and hydrophobicity [48]. C2 and C3 were positively predicted by the proportion of cropland use and utilized by more activated microbial species, but FI was negatively predicted by the proportion of built-up land use except in winter, revealing that domestic wastewater was purified to a better quality before discharge [3]. Forestland and cropland were used to positively predict DOC and C1 and had the distinctive feature of a high molecular weight in summer. C2 was still regulated by microbial metabolites (BIX) in all seasons (Figure 9), due to large molecular size; oxygen-rich WEOM in soil was scoured into the water course by stormwater after heavy corrosion [49].

There was a positive correlation between DOC and the proportion of cropland in summer (*p* < 0.01) (Figure 9a–d), indicating that DOC concentration was seriously affected by land-cultivated activities, which may be due to the soil microbial products and humics transportation caused by heavy precipitation which facilitated N, P-nutrients to be absorbed by microbes and phytoplankton.

The linear correlation diminished in other seasons, but still there was susceptibility to soil weathering and erosion by river water that resulted in the loss of organic and nutritious elements to feed or stimulate bacteria [50]. The result is contrary to previous observations, in which it was found that agricultural land lowered the proportion of protein-like compounds [15].

C1 as delineated above and humic-like DOM had homology with protein-like substances which originated from instream or in situ microbial degradation of soil organic detritus during reclamation, but the correlation was not so obvious as between C2 and agricultural proportion. In northern German plains and large rivers in western China, DOM from agricultural headwater streams contained a higher proportion of structurally complex, humic substances than DOM from nearby forested streams [20,51]. In this study, however, cropland and forested land use showed DOM with similar qualities in both rivers all year round, and with no relevant effect of humic substances of complex structure most of the time, and sometimes an inverse influence from two types of land use. CDOM was negatively predicted by built-up land use and the seasonal influence had no significant effect. It was revealed that pollutants were removed through sewage disposal systems and pollution was relieved before being released to rivers and the purification system was sustained in a better situation (Figure 9a–d) [13].

In general, intensive human activities in cities can increase protein-rich DOM inputs, and the discharge of domestic sewage may stimulate microbial and phytoplankton activities [52]. As the content of CDOM (a_335_), protein-like substances should increase with the expansion of built-up areas. The discharge of DOM from industrial wastewater into the environment will not solely affect the absorption of nutrients by bacteria, plankton and algae, but also change the level of DOM and the physical and chemical properties of water [2]. However, the relationship between built-up areas and DOM or CDOM was inconsistent with the above description. On the contrary, the HIX value was positively correlated with built-up areas, which can be related to the decreasing proportion of C2 in DOC in densely populated areas. The quantity of macromolecules of complex structure incremented in DOC comparing to other land-use type regions. These results coincide with the fact that the planktonic bacteria may produce humus-like substances in lakes after modifying protein-like substances, therefore increasing the proportion of humus-like substances and decreasing the proportion of protein-like substances [41].

The aromaticity of DOC in autumn was influenced positively by cropland use, had a small molecular size, and it can be speculated that it was associated with soil erosion in the riparian zone [53]. Forestland can produce more DOM, and the molecular weight in soil is larger [52]. There was a positive correlation between DOC and the proportion of forestland use (*p* < 0.05) (Figure 9a–d), indicating that rivers flowing through forests carried high OC load and had a high molecular weight in autumn and winter [18,54]. However, low or high molecular weight in spring and summer, respectively, was the result of non-point source pollution and related to protein-like and humic-like substance input [8]. The positive correlation between C2 and forestland only occurred in spring (r = 0.612, *p* < 0.05), explaining the effect of forestland on protein substances in rivers (Figure 9a) due to the decay of lignin in soil in producing microbial compounds after the last severe winter. This phenomenon is a sign of homology of fluorescent components (C1 vs. C2, sourced from forested land region). The positive correlation between C2 and cropland also changed seasonally (*p* < 0.01), and the correlation gradually decreased from spring to winter (Figure 9a–d), which indicates that agricultural activities made a great contribution to the formation of protein-like substances.

Agricultural land has smaller aggregates, more soil organic matter was exposed to microbial decomposition, and fewer organo-mineral associations reduced the physical protection of soil organic matter, making this area more sensitive to soil disturbance with agricultural practices [55]. The application of fertilizer containing N and P promoted microbial activity and algal growth or eutrophication of water in the aquatic environment, resulting in a high conversion of organic matter precursors, primary producer exudate and microbial metabolism in water [56,57].

In spring and summer, BIX, a_355_ and a_260_ were positively correlated with the proportion of cropland (*p* < 0.01) (Figure 9a,b), mainly because anthropogenic input of organic fertilizer in farmland and soil ploughing increased nitrogen and phosphorus and activated microbial reactions and DOM autochthonous sources, making it easier for more hydrophobic CDOM and other substances to be loaded in the surrounding water [39,52]. SUVA_254_ was better aligned by RDA2 in winter and it was positively predicted by grassland.

In Duliujian River, DOC, C1, C2, S_R_, a_355_ and a_260_ were aligned better with RDA1 in all four seasons, in which DOC and C1 were positively predicted by the proportion of forestland and of cropland use in spring and summer, and had good hydrophobicity. It is well known that allochthonous sources of DOM in the coastal ocean derive mainly from the degradation of terrestrial vegetation (e.g., native forests and scrublands) and soil organic matter from tributary hydrographic basins [58]. This allochthonous organic matter consists of a complex mixture of higher molecular weight and more refractory compounds [59].

**Figure 10:**
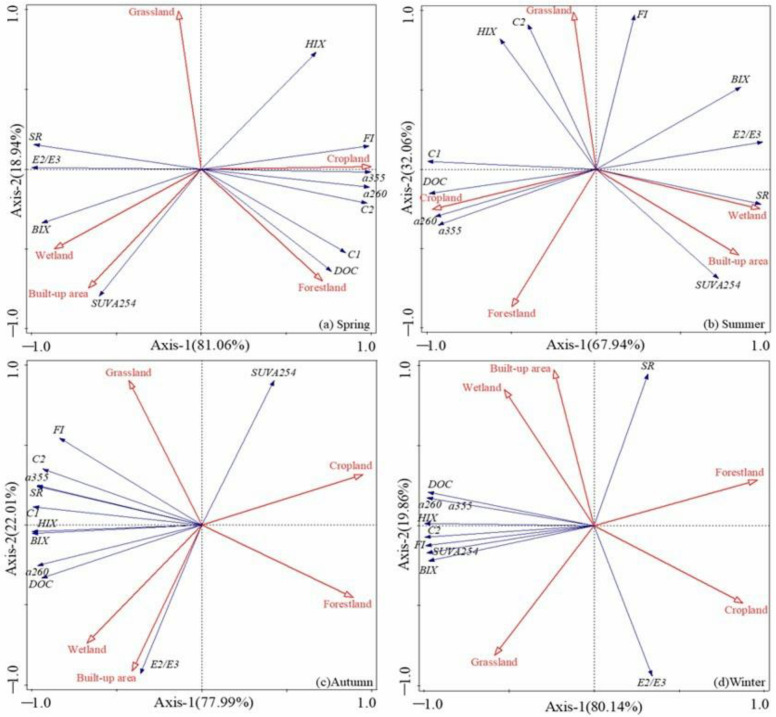
Redundancy analysis (RDA) results of the correlation between land-use types and UV absorption coefficients, fluorescence intensity and fluorescence characteristic parameters (HIX, BIX and FI) in Duliujian River.

During this time period, a_355_ and a_260_ were positively correlated with the proportion of cropland (R^2^ = 0.378, *p* < 0.05; R^2^ = 0.325, *p* < 0.05) (Figure 10a,b), which was similar to that of Haihe River, mainly due to the increase of CDOM content (a_355_) caused by agricultural planting activities. In spring, C2 also showed a similar trend to cropland proportion (R^2^ = 0.947, *p* < 0.01) (Figure 10a), indicating that plowing activities may bring DOM signals containing proteins of high molecular size. Unlike Haihe River, BIX had a strong positive correlation with the proportion of wetland and of built-up land use in Duliujian River (R^2^ = 0.77; *p* < 0.05) (Figure 10a), and the correlation decreased with seasonal shifts, caused by the increase in the relative amount of protein-like to humic-like compounds in DOC.

The chemical engineering industry in the built-up area had a great impact on the DOM of the Duliujian River basin. Appropriate temperatures and slow water flow created a comfortable breeding environment for microbial and planktonic species. Photobleaching, sedimentation, mineralization, flocculation etc. become the controlling factors on the outset from particulate materials to large molecules and small ones, and eventually inorganic compounds in spring [60]. DOC in autumn and winter in Duliujian River was predicted negatively by agricultural land use, which was mainly sourced from microbial metabolites. It was characteristic of hydrophobicity and humification, positively predicted by the proportion of wetland and built-up land (Figure 10c,d). C2 was positively predicted by the proportion of grassland use and susceptible to strong biological metabolism, hydrophobic and aromatic in winter and with higher molecular size in spring. SUVA_254_, S_R_, FI and C2 were better aligned by RDA2 and positively predicted by the proportion of grassland use, similarly to Haihe River.

Grassland probably prolonged water residence time in the surface soil horizon and increased the quantity of organic carbon transported to rivers [18].

S_R_ in winter was negatively influenced by grassland; the molecular size of DOM increased due to decomposed plant detritus being carried into water courses after a series of photochemical and microbial alterations [61]. Differently from Haihe River watershed, C2 and FI were connected with the proportion of grassland use especially in summer, autumn and winter; this was due to soil crop rotation and the soil plow layer being exposed to microbial decomposition, accompanied by soil organic matter (WEOM, water extractable organic matter) that was flushed out into water in summer. This result induced homology to components and the phenomenon of homogenization (BIX vs. HIX), especially in autumn and winter [53]. Vegetation type and the biogeochemical pattern of OC transformation also had an important influence on changes in the chemical composition, structure and nature of OC except for the chemical recalcitrance or organo-mineral associations [62].

The RDA analysis illustrates the effects of the multivariate structure and highlights the role that the extensive cover of native forest, grassland and wetland had on the concentration of CDOM and humic components in the Haihe and Duliujian River watershed, and its divergence from built-up land and agricultural land use. The spatial variation in concentrations could be subjected to degradation and transformation processes undergone by fluorescent components along the water flow in rivers. Soil processes were involved in intervening in quality and quantity changes of components in the riparian zone as well as water quality. Change of land cover caused by human activity (livestock stomping, soil tillage, ploughing, drainage) altered the stream water DOM quality and quantity by strengthening the export of microbially-processed soil compounds to streams, yet the instream processes due to nutrient enrichment strengthened the microbial signatures.

The combination of geospatial data and organic matter measurement achieved an interdisciplinary integration for exploring the effect of land use on river organic matter sources, composition and content. The limitation is that there is no comparison between different geographical results on a large scale and a lack of soil property analyses of riparian buffering zones.

## 5. Conclusions

Spatiotemporal variations of CDOM in a mixed land use river were investigated to reveal abundant information for water quality management in northern China. The amount and composition of CDOM are closely related to pollution sources. A high and stable quantity of humic-like and protein-like substances confirmed the influence of non-point source pollutants throughout the river, which should be considered a priority for water quality restoration due to their prevalent impacts and unregulated status. The CDOM data demonstrate that the pollution characteristics of Haihe and Duliujian River were driven and impacted by both land-use and seasonal factors. Fluorescent components reflected the pollution sources which were not indicated by water parameters. By applying the EEM-PARAFAC model, the fluorescent materials were identified as three components. The humification degree of DOM in the two rivers was relatively low, and the source was mainly autochthonous, generated by the degradation of microorganisms and phytoplankton. DOM change was controlled by an allochthonous humic-like and autochthonous protein-like double change. The CDOM content of both rives and hydrophobicity in Haihe River were affected by cropland, resulting from nitrogen and phosphorus in chemical fertilizers needed in cultivation land which can stimulate the growth of microorganisms and increase the content of CDOM. The higher amount of autochthonous protein-like substances in Haihe River in spring was influenced by forestland. DOM was inversely related to built-up areas, which may be related to urban water purification systems. However, BIX of Duliujian River was greatly affected by built-up areas, and the discharge of wastewater may increase the autochthonous characteristics of DOM. For water quality restoration, control of C2 and C3 should be a primary goal, with a particular concentration of efforts in the middle stream area of Haihe River. The comprehensive analysis of DOM spectral characteristics and land-use types provides a new idea for the study of the watershed carbon cycle, which is conducive to further explorations of the impact of urban land-use types on water environment.

## Figures and Tables

**Table 1:** The mean values of water chemical parameters of sampling sites in different seasons.

Parameters	Units	Spring		Summer		Autumn		Winter	
		H1–H12	D1–D3	H1–H12	D1–D3	H1–H12	D1–D3	H1–H12	D1–D3
pH		8.56 ± 0.84	8.74 ± 0.33	8.33 ± 0.484	8.64 ± 0.35	8.44 ± 0.30	8.45 ± 0.35	8.16 ± 0.31	8.33 ± 0.01
T	°C	15.03 ± 4.57	16.33 ± 4.87	28.27 ± 1.81	27.93 ± 1.35	16.24 ± 6.70	16.37 ± 7.68	4.48 ± 1.56	2.41 ± 0.74
EC	ms·cm^−1^	2.30 ± 4.04	22.54 ± 22.40	1.77 ± 2.98	6.66 ± 14.65	1.20 ± 0.96	1.59 ± 0.74	1.81 ± 1.61	1.07 ± 0.29
DO	mg·L^−1^	9.92 ± 1.81	9.85 ± 1.37	6.76 ± 3.08	7.78 ± 1.88	10.04 ± 3.07	9.88 ± 2.28	14.17 ± 2.45	14.01 ± 0.05
Chl-a	μg·L^−1^	20.76 ± 23.58	27.90 ± 17.80	40.44 ± 49.48	34.98 ± 22.17	53.91 ± 47.99	34.53 ± 6.75	41.34 ± 78.37	18.59 ± 3.50
Cl^−^	mg·L^−1^	435.01 ± 1128.04	2975.11 ± 6354.72	377.76 ± 942.44	2260.73 ± 5845.12	209.14 ± 321.58	271.21 ± 184.17	595.26 ± 694.18	220.87 ± 102.84
NO_3_^−^	mg·L^−1^	0.58 ± 0.46	0.42 ± 0.49	0.78 ± 0.54	0.34 ± 0.25	1.66 ± 0.47	1.78 ± 0.90	1.53 ± 0.27	2.17 ± 0.11
SO_4_^2−^	mg·L^−1^	150.98 ± 225.49	538.14 ± 743.76	155.02 ± 185.52	454.289 ± 714.43	140.91 ± 80.63	225.14 ± 53.04	190.59 ± 127.07	188.80 ± 44.27
HCO_3_^−^	mmol·L^−1^	3.03 ± 1.24	3.38 ± 1.38	2.88 ± 1.04	3.42 ± 0.57	3.53 ± 0.91	5.77 ± 4.60	3.68 ± 0.85	4.28 ± 0.26
CO_3_^2−^	mmol·L^−1^	0.06 ± 0.07	0.05 ± 0.03	0.06 ± 0.08	0.09 ± 0.05	0.05 ± 0.05	0.15 ± 0.34	0.02 ± 0.02	0.02 ± 0.00
Na^+^	mg·L^−1^	286.27 ± 646.49	1697.97 ± 3358.61	265.47 ± 594.97	1344.90 ± 3258.86	156.53 ± 198.58	208.12 ± 112.91	385.90 ± 415.47	176.10 ± 70.52
K^+^	mg·L^−1^	20.74 ± 47.54	57.29 ± 116.48	19.06 ± 45.29	50.76 ± 113.04	9.25 ± 8.09	8.95 ± 3.10	16.39 ± 15.22	8.08 ± 1.84
Mg^2+^	mg·L^−1^	45.47 ± 75.81	257.14 ± 437.22	44.93 ± 69.68	192.98 ± 419.24	39.50 ± 28.77	58.18 ± 15.56	68.51 ± 56.17	51.06 ± 12.20
Ca^2+^	mg·L^−1^	52.30 ± 38.50	127.30 ± 141.67	62.57 ± 29.37	119.29 ± 145.65	77.02 ± 32.69	99.40 ± 11.88	91.21 ± 30.01	98.51 ± 8.52

**Table 2:** Characteristics of the three fluorescence components identified in this study compared with those previously described.

Components	Ex_max_/Em_max_	Description and Source	References
C1	250(290)/440; 255/412;225/449	Terrestrial and marine humic-like materials	C1: <250(310)/416 [36]; C1: <250/448 [35]; Peak A: 230–260/380–460, Peak C: 290–310/370–420 [4]
C2	230(280)/345; 225(275)/344	tryptophan-like	C2: 220(285)/348 [34]; C4: <250/350 [16]; Peak T: 225–230(275)/340–350 [4];
C3	225(260)/301 225(265)/325;	Autochthonous tyrosine-like	C4: 230(270)/306 [37]; C8: 275/304 [35]; C3: 220(270)300 [34];
